# Acute kidney injury in idiopathic nephrotic syndrome of childhood is a major risk factor for the development of chronic kidney disease

**DOI:** 10.1080/0886022X.2016.1277743

**Published:** 2017-01-17

**Authors:** Afshan Yaseen, Vina Tresa, Ali Asghar Lanewala, Seema Hashmi, Irshad Ali, Sabeeta Khatri, Muhammed Mubarak

**Affiliations:** Department of Pediatric Nephrology and Histopathology, Sindh Institute of Urology and Transplantation (SIUT), Karachi, Pakistan

**Keywords:** Acute kidney injury, children, chronic kidney disease, idiopathic nephrotic syndrome, Pakistan

## Abstract

**Background:** Acute kidney injury (AKI) is an important complication of idiopathic nephrotic syndrome (INS) and is associated with adverse outcomes, especially the development of chronic kidney disease (CKD). We aimed to determine the clinical profile of children with INS who developed AKI and its short-term outcome.

**Material and methods:** This prospective study was conducted from March 2014 to October 2015. A total of 119 children of INS (age: 2–18 years) fulfilling the pediatric RIFLE criteria for the diagnosis of AKI were enrolled and followed up for 3 months to determine the outcome. Factors predisposing to CKD were studied.

**Results:** The mean age at presentation was 8.8 ± 3.59 years and males were 74 (62.2%). At presentation, 61 (51.3%) children were in Risk category, 43 (36.1%) in Injury category, and 15 (12.6%) in Failure category. Most of them (41.2%) had steroid-resistant nephrotic syndrome (SRNS) and focal segmental glomerulosclerosis (FSGS) on histopathology (33.6%).

Infections were the major predisposing factor for AKI in 67 (56.3%) cases. Drug toxicity was the next common, found in 52 (43.7%) children. A total of 65 (54.6%) children recovered from AKI, while 54 (45.4%) did not. CKD developed in 49 (41.2%) non-recovered cases and 5 (4.2%) children succumbed to acute illness. SRNS, cyclosporine use, FSGS on histology, and drug toxicity were significant factors associated with the development of CKD.

**Conclusion:** AKI associated with INS is a reversible condition in most cases but it can progress to CKD, especially among those who have SRNS, FSGS, and drug toxicity.

## Introduction

Acute kidney injury (AKI) is an important and alarming complication of idiopathic nephrotic syndrome (INS).^1^^,^^2^ Burden of AKI in INS has increased recently, with reported incidence found to be 50.9% in United States.^3^ AKI, usually reversible, has been observed in children with normal or minimally altered glomeruli on renal biopsy. It may occur either at the time of presentation or much later.^4^ The etiology of AKI in children with INS varies, and includes pre-renal failure, acute tubular necrosis (ATN), drug toxicity, like calcineurin inhibitor (CNI), angiotensin converting enzyme (ACE) inhibitor, and angiotensin receptor blocker (ARB)-induced toxicity, sepsis, renal vein thrombosis, peritonitis, and interstitial nephritis.^5^ Data on biopsy findings have rarely been reported in children as well as in adults. Kidney biopsies performed in children during an episode of AKI in INS, most often demonstrate no specific cause for AKI other than underlying disease; ATN and interstitial nephritis were found in some children.^3^ Patients with INS have several risk factors for ATN. They may be intravascular volume depleted due to the decreased oncotic pressure that accompanies hypoalbuminemia in NS. Intravascular volume depletion is also often exacerbated by the frequent use of diuretics in this population. Many medications used in the treatment of NS, such as cyclosporine and tacrolimus, can also potentially alter renal perfusion.^6^ AKI is associated with adverse outcomes in hospitalized children and can progress to chronic kidney disease (CKD).^3^^,^^7^^,^^8^ Most pediatric studies on AKI with INS are limited to developed countries. We aimed to prospectively determine clinical profile and outcome at three months follow-up of AKI with INS in children.

## Methods

### Study population and case definition

This prospective observational study was conducted at the Pediatric Nephrology Department of Sindh Institute of Urology and Transplantation (SIUT) from March 2014 to October 2015. The study was conducted in accordance with tenets of Declaration of Helsinki. All children with INS between 2 and 18 years of age fulfilling the modified pediatric RIFLE criteria were enrolled for the study and followed up for three months. All children with secondary NS, acute-on-chronic renal failure, age less than 2 years at the time of diagnosis of INS and base line estimated glomerular filtration rate (eGFR) less than 90 ml/min/1.73 m^2^ were excluded. INS was defined as the presence of edema, serum albumin <25 g/L, proteinuria >40 mg/m^2^/hour, or urine protein creatinine ratio >200 mg/mmol. The method of laboratory determination of serum creatinine (S.Cr) was done by the Jaffe method. eGFR was calculated by the Schwartz equation (K × height/S.Cr).^9^ For baseline eGFR, lowest value of S.Cr in 3 months preceding study enrollment was taken. If no previous S.Cr was available, then patient was assumed to have baseline eGFR of ≥90 ml/min/1.73m^2^.

AKI patients were diagnosed using the modified pediatric RIFLE score through the use of eGFR criteria only (stage R: eGFR decreased by 25%, stage I: eGFR by 50%, stage F: eGFR by 75% or less than 35 ml/min/1.73m^2^).^10^ Steroid sensitive nephrotic syndrome (SSNS), steroid-dependent nephrotic syndrome (SDNS) and steroid-resistant nephrotic syndrome (SRNS) were classified as per standard definitions.^11^ Complete recovery was defined as eGFR ≥90 ml/min/1.73 m^2^ by the Schwartz equation. CKD was defined as persistent deterioration in renal functions for more than 3 months. Infection, sepsis, and severe sepsis were defined according to surviving sepsis campaign guidelines.^12^

### Study endpoint and follow-up

The primary endpoint was normalization of renal functions. Patients were followed for 3 months after an episode of AKI to see the outcome (CKD/Death/Recovered). After patients improved clinically, they were followed up in the outpatient clinic and S.Cr was done according to clinical condition of the patients.

### Statistical analysis

All data were analyzed by using SPSS version 16 (SPSS, Inc., Chicago, IL). Normal or near normally distributed variables were reported as means ± standard deviation (SD). Frequency and percentage were calculated for qualitative variables. Categorical data were tested using the Student *t*-test, Chi-square test, or Fisher exact test. All statistical tests were two-tailed. *p* Value ≤0.05 was considered statistically significant.

## Results

### Demographics and clinical profile

Overall, 119 children, with INS were studied for AKI during the study period. The mean age of the entire cohort was 8.8 ± 3.59 years (range: 2.2–16 years). There were 74 (62.2%) males and 45 (37.8%) females. Baseline clinical and biochemical characteristics of children with AKI in INS are depicted in Table 1. At presentation, 61 (51.3%) children were in the Risk category, 43 (36.1%) in Injury category, and 15 (12.6%) in Failure category.

**Table 1.** Baseline clinical and biochemical characteristics of children with acute kidney injury in idiopathic nephrotic syndrome (*n* = 119).

	Numbers (*n*)	Percentage
Characteristics		
Diarrhea	32	26.9
Vomiting	20	16.8
Fever	61	51.3
Oligoanuria	42	35.3
Hypertension	12	10.1
Hypotension	7	5.9
Dehydration	11	9.2
Edema	35	29.4
Shock	6	5.0
Anemia	83	69.7
Leukocytosis	53	44.5
Acidosis	80	67.2
Hypoalbuminemia	92	77.3
Proteinuria	95	79.8
Microscopic hematuria	33	27.7
Histopathology		
Biopsy not done	27	22.7
FSGS	40	33.6
MCD	24	20.2
MesPGN	12	10.1
MCGN	6	5.0
Membranous nephropathy	7	5.9
IgA N	1	0.8
IgM N	2	1.7

FSGS: focal segmental glomerulosclerosis; IgAN: IgA nephropathy; IgMN: IgM nephropathy; MCD: minimal change disease; MesPGN: mesangioproliferative GN; MCGN: mesangiocapillary GN.

### Etiology of AKI

**Figure 1.**
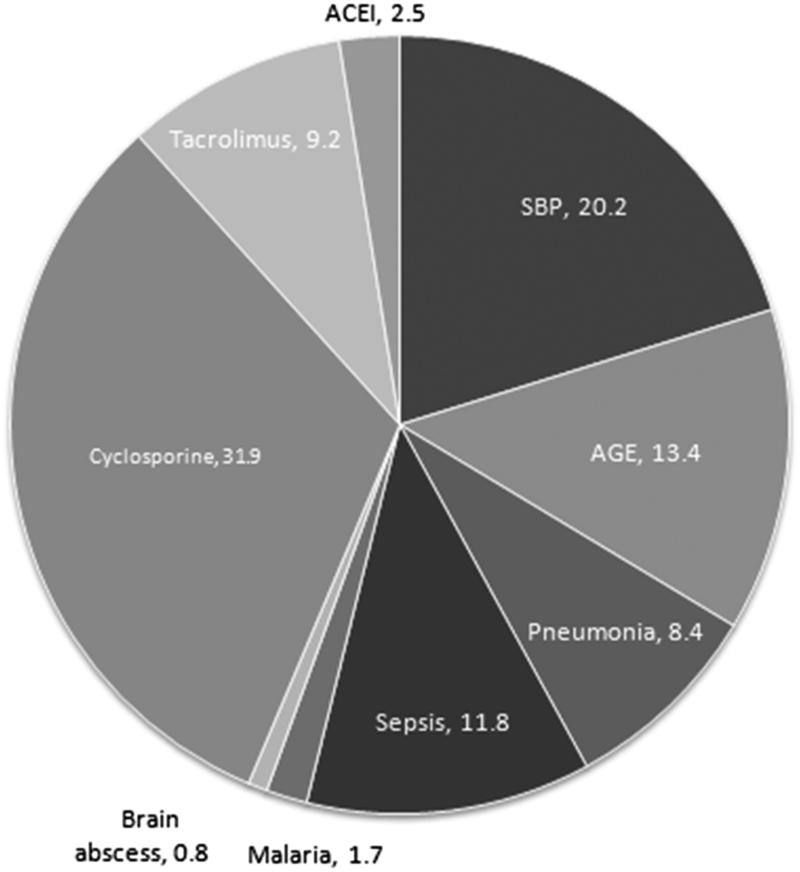
Most likely etiological factors leading to acute kidney injury in 119 children with idiopathic nephrotic syndrome. ACEI: angiotensin converting enzyme inhibitor; AGE: acute gastroenteritis; SBP: spontaneous bacterial peritonitis.

The most probable etiologic factors predisposing to AKI development in nephrotic children are shown in Figure 1. Among 119 children, infection was found as the most common cause (*n* = 67, 56.3%). Types of infections included: spontaneous bacterial peritonitis (SBP) in 24 (20.2%), acute gastroenteritis (AGE) in 16 (13.4%), sepsis in 14 (11.8%), pneumonia in 10 (8.4%), malaria in 2 (1.7%), and brain abscess in 1 (0.8%). Drug toxicity was the second most common cause, found in 52 (43.7%) cases. It included cyclosporine in 38 (31.9%), tacrolimus in 11 (9.2%), and ACE inhibitors in 3 (2.5%) cases.

### Short-term outcome

An analysis of the short-term outcome of AKI in INS showed that slightly more than half of children (65: 54.6%) recovered from AKI. Of the remaining 54 (45.4%) who did not, 49 (41.2%) developed various degrees of CKD and 5 (4.2%) patients died. A total of 17 (14.3%) patients progressed to CKD stage 2, 18 (15.1%) to CKD stage 3, 10 (8.4%) to CKD stage 4 and 4 (3.4%) to CKD stage 5/ESRD. The cause of death was septic shock in 5 (4.2%) patients. Out of these 5 patients, 2 were in the Risk category and 3 had developed Failure according to modified pediatric RIFLE criteria at the time of presentation.

### Predictors of AKI in INS progressing to CKD

The factors responsible for the progression of AKI to CKD in children with INS are shown in Table 2. Children with SRNS, FSGS on histopathology, cyclosporine use and AKI secondary to drug toxicity, were significantly more prone to progress to CKD.

**Table 2.** Factors responsible for chronic kidney disease (CKD) development in children with idiopathic nephrotic syndrome.

Risk factors	Recovered *n*(%)	CKD *n*(%)	Odds ratio (95% CI)	*p*-Value
SRNS	21 (42.9)	28 (57.1)	3.11 (1.45–6/66)	0.003
Cyclosporine use	21 (46.7)	24 (53.3)	2.24 (1.04–4.78)	0.036
FSGS	14 (35.0)	26 (65.0)	4.52 (2.00–10.17)	<0.001
Drug Toxicity	24 (47.1)	27 (52.9)	2.3 (1.08–4.87)	0.029

CI: confidence interval; FSGS: focal segmental glomerulosclerosis; SRNS: steroid resistant nephrotic syndrome.

## Discussion

To the best our knowledge, this is the largest single center and first prospective study to date examining the incidence of AKI in children with INS and their short-term outcome in a developing country from South Asia. We used pRIFLE classification to diagnose AKI,^10^ as it was found to be the most sensitive classification system for the diagnosis of AKI.^13–15^ In this study, we found most patients of AKI in the Risk category (*n* = 61, 51.3%), as reported previously by Renault et al.^3^

In this study, males were predominant (*n* = 74, 62.2%) and most children belonged to 5–10 years age group (*n* = 48, 40.3%). These results are comparative to a number of previous studies on this subject.^1^^,^^3^^,^^16^ Most children had a history of diarrhea, vomiting, dehydration, and shock predisposing to pre-renal failure. Citing similar sporadic case reports dating from 1934, Chamberlain et al. concluded that intravascular volume depletion arising either spontaneously or following therapeutic interventions triggered a marked decline in GFR and oliguria.^17^ ATN was thought to supervene when renal ischemia was severe in some cases. In their analysis, these investigators relied primarily on published reports of low plasma volume in NS to support the hypothesis that hypovolemia was operative.^18^ It is noteworthy that in these cases, clinical evidence for volume contraction was not described. Interestingly, infections (56.3%) were identified as the major underlying predisposing factor for AKI. Drug toxicity was the second most common etiology found in 43.7% cases and most (31.9%) had cyclosporine-induced AKI. A recent study also showed same risk factors for AKI.^3^^,^^16^ We also found that 49 (41.2%) children developed CKD at three months and 5 (4.2%) children died. There is no study available to follow outcome in INS with AKI, but a study published in 2001 showed that ∼30% of children and adults with INS have a significant reduction in GFR before AKI.^19^ Recently, there has been increasing recognition that AKI is a risk factor for the development and progression of CKD in children.^20^^,^^21^

This study has several strengths. This is one of the largest studies in the pediatric population on this subject from a developing country using pRIFLE criteria for the diagnosis and categorization of AKI. The prospective nature of the study and follow-up of patients, albeit for short period, for determining outcome are also added attributes in a resource-constrained setting.

There are certain limitations in the study too which must be kept in mind when interpreting the results from this study. These include the origin of study from a single center, lack of biopsies in all patients, and a short follow-up period. We also did not analyze the trend of GFR in the cohort to demonstrate progression or stability of GFR in these children due to short follow-up period. In spite of these limitations, we believe that our study is one of the largest studies on the incidence of AKI in children with INS from any single center in a developing country. This will help increase awareness of the nephrologists about this complication, which can lead to CKD in many children. The increased awareness coupled with attention to hygiene and avoidance of potentially nephrotoxic medications may possibly be helpful in declining progression of AKI to CKD.

## Conclusions

AKI associated with INS in most cases is a reversible condition but it can progress to CKD, especially in those with SRNS, FSGS and drug toxicity. Further, large-scale, multi-center studies with long-term follow-up is needed to identify risk factors and prognosis of AKI in INS.
